# Cancer therapy and cardiotoxicity: The need of serial Doppler echocardiography

**DOI:** 10.1186/1476-7120-5-4

**Published:** 2007-01-25

**Authors:** Maurizio Galderisi, Francesco Marra, Roberta Esposito, Vincenzo Schiano Lomoriello, Moira Pardo, Oreste de Divitiis

**Affiliations:** 1 Division of Cardioangiology with CCU of Department of Clinical and Experimental Medicine, Federico II University Hospital, Naples, Italy

## Abstract

Cancer therapy has shown terrific progress leading to important reduction of morbidity and mortality of several kinds of cancer. The therapeutic management of oncologic patients includes combinations of drugs, radiation therapy and surgery. Many of these therapies produce adverse cardiovascular complications which may negatively affect both the quality of life and the prognosis. For several years the most common noninvasive method of monitoring cardiotoxicity has been represented by radionuclide ventriculography while other tests as effort EKG and stress myocardial perfusion imaging may detect ischemic complications, and 24-hour Holter monitoring unmask suspected arrhythmias. Also biomarkers such as troponine I and T and B-type natriuretic peptide may be useful for early detection of cardiotoxicity. Today, the widely used non-invasive method of monitoring cardiotoxicity of cancer therapy is, however, represented by Doppler-echocardiography which allows to identify the main forms of cardiac complications of cancer therapy: left ventricular (systolic and diastolic) dysfunction, valve heart disease, pericarditis and pericardial effusion, carotid artery lesions. Advanced ultrasound tools, as Integrated Backscatter and Tissue Doppler, but also simple ultrasound detection of "lung comet" on the anterior and lateral chest can be helpful for early, subclinical diagnosis of cardiac involvement. Serial Doppler echocardiographic evaluation has to be encouraged in the oncologic patients, before, during and even late after therapy completion. This is crucial when using anthracyclines, which have early but, most importantly, late, cumulative cardiac toxicity. The echocardiographic monitoring appears even indispensable after radiation therapy, whose detrimental effects may appear several years after the end of irradiation.

## Background

In the last decade cancer therapy (CT) has shown a terrific progress leading to an important reduction of morbidity and mortality of several kinds of cancer. The therapeutic management of patients with cancer includes multiple combinations of drugs, radiation therapy and surgery. Many of these therapies produce potential adverse cardiac reactions which can negatively affect the quality of life as well as the prognosis of oncologic patients.

In this view, the early detection of cardiotoxicity due to CT is a critical issue in the clinical setting, in order to interrupt or modulate appropriately CT and even to sustain ventricular performance by cardiac drugs. The traditional screening of patients with cancer includes cardiologic examination, and both EKG and Doppler echocardiography at rest. The monitoring of cardiovascular toxicity might be more accurate by using endomyocardial biopsy [1] which is, however, invasive and not completely safe. For several years the most common noninvasive method of monitoring cardiotoxicity has been represented by radionuclide ventriculography. Other cardiac tests as effort EKG and stress myocardial perfusion imaging may be used to detect ischemic myocardial complications, while 24-hour Holter monitoring becomes helpful to unmask suspected arrhythmias. Also biomarkers such as troponine I and T [2,3] and B-type natriuretic peptide (BNP and NT-proBNP) [4,5] may be useful for early diagnosis of cardiotoxicity.

During time echocardiography is emerged as the choice test for noninvasive evaluation of cardiac disease due to cancer therapy. This tool is essential for the evaluation of left ventricular (LV) systolic and diastolic dysfunction, pericardial disease, myocardial damage and detailed information of valvular heart disease. Nevertherless, Doppler echocardiographic examination is routinely planned only at the beginning of CT in order to document a normal LV systolic function. Further echocardiographic controls during CT are performed only as a consequence of the onset of cardiac symptoms and/or signs, in particular following the administration of recognized cardiotoxic drugs or the radiation therapy. On these grounds, these review attempts to demonstrate the clinical need of serial Doppler echocardiographic evaluation and the potential impact of advanced ultrasound technologies in patients undergoing CT.

## Cardiac toxicity of chemotherapic agents and radiation therapy

Cardiovascular toxicity gives its expression in silent (pre-clinical) or overt (clinical) events. Pre-clinical toxicity may be diagnosed by histopathological or biochemical techniques and, even, by detailed imaging techniques. The grading system proposed by the World Health Organization to standardize the report of adverse effects due to CT does not consider laboratory or modern imaging changes [6] while the more comprehensive system of the National Cancer Institute, including all the important clinical and laboratory changes, should be updated.

Cardiovascular effects associated with CT agents are listed in Table 1. ***Anthracyclines ***(Doxorubicin, Daunorubicin, Epirubicin, Idarubicin), the best studied anticancer drugs, widely used for several hematologic and solid tumors, have a recognized cardiotoxicity, which may be acute and/or late. Acute cardiotoxicity develops producing non-specific EKG abnormalities but sometimes also cardiac symptoms. Late cardiotoxicity is cumulative (e.g., doses of doxorubicin > 550 mg/m^2^), dose-related and may determine LV systolic dysfunction until congestive heart failure (CHF). The mechanism underlying this dysfunction is consequence of a direct myocardial damage induced by the formation of free radicals [7]. It has been estimated a very high mortality rate in patients developing anthracycline-related CHF [8] but this rate may be strongly reduced by early diagnosis and therapy. ***Alkylators ***(e.g., Busulfan, Cyclophosphamide, Cisplatin, Mytomicin), commonly used for several solid tumors and lymphomas, may induce cardiac complications including heart failure, myocarditis and pericarditis [9]. The mechanism underlying the damage is related to endothelial and cardiomyocyte injury mediated by a toxic metabolism [10]. The total dose of an individual course, more than the cumulative dose, appears to be the best predictor of cardiotoxicity of cyclophosphamide [11]. Cisplatin can determine late cardiovascular complications (arterial hypertension, myocardial ischemia and infarction), even 10–20 years after the remission of metastatic cancer [12]. Reported cardiotoxic effects of ***Antimetabolities ***(5-fluorouacil, capecitabine), widely used in the treatment of solid tumors, may produce ischemic syndrome (angina pectoris and myocardial infarction), arrhythmias and cardiomyopathy [13]. Of note, the pro-ischemic mechanisms of 5-fluorouracil include also a very peculiar induction of acute coronary vasospasm [14]. ***Antimicrotubule Agents ***(Paclitaxel, Vinca Alkaloids), applied to the treatment of several solid tumors, may induce arrhythmias, thrombosis and myocardial ischemia [15]. Of note, Paclitaxel has been recently used to coat coronary stents following percutaneous coronary angioplasty. High-dose of ***Cytokines ***(Interleukin-2, Denileukin, Interferon-α), used for treatment of metastatic renal carcinoma and melanoma, can results in arterial hypotension, vascular leak syndrome, arrhythmias, cardiomyopathy and vascular thrombosis [16]. The treatment by ***All-trans Retinoic Acid***, a vitamine A derivative approved for acute promyelocytic leukemia, induces a syndrome characterized by fever, dyspnoea, arterial hypotension and pericardial/pleural effusion within the first 21 days [17], with depression of LV function in 17% of the cases [18]. ***Arsenic Trioxide***, used for refractory or relapsed acute promyelocitic leukemia, provokes QT-interval prolongation and arrhythmias in more than 50% of treated patients but can determine also fluid retention (pleural and pericardial effusion) [19]. ***Imatinib Mesylate***, a specific inhibitor of BCR-ABL tyrosine kinase used for chronic myelogenous leukemia and other malignancies, is associated with a significant incidence of fluid retention [20]. The most common cardiac adverse effect of therapy by ***Etoposide***, mainly used for refractory testicular tumors and small-cell lung carcinoma, is arterial hypotension but myocardial ischemia may be observed [21]. ***Monoclonal Antibodies ***(Alemtuzumab, Bevacizumab, Cetuximab, Rituximab), modernly applied for treatment of some hematologic malignancies and solid tumors, have toxicity profiles specific to the blocked receptors but all produces arterial hypotension [22]. In this category, Trastuzumab, recombinant IgG1 monoclonal antibody binding selectively to the human epidermal growth factor receptor 2 protein and approved for treatment of breast cancer that overexpresses HER2, has a recognized incidence of congestive heart failure [23].

**Table 1:** Adverse cardiovascular effects of cancer therapy

**Adverse cardiac effect**	**Therapy**
Heart failure	Anthracycline, Mitomycin, Cyclophosphamide, Cisplatin, Trastuzumab, Alemtizumab
Pericardial/Pleural effusion	Cyclophosphamide, Cytarabine, Imatinib, Thalidomide, Trans-retinoic acid, Busulfan, Radiation therapy
Myocardial ischemia	Cisplatin, Vinca Alkaloids, Capecitabine, Interleukin-2 Bevacizumab, 5-Fluotouracil, Radiation therapy
Arterial hypertension	Cisplatin, Bevacizumab, Interferon-α
Arterial hypotension	Etoposide, Talidomide, Paclitaxel, Alemtuzumab, Cetuximab, Rituximab, Transretinoic Acid, Interleukin-2, Interferon-α
Myocarditis	Busulfan, Cyclophoshamide, Radiation Therapy
Bradycardia	Thalidomide, Paclitaxel
QT prolongation	Arsenic Troxide
Thromboembolis	Bevacizumab, Paclitaxel

The ***radiation therapy ***is applied for CT of lymphoma, breast cancer, thymoma, high and low respiratory ways, esophageal and gastric lesions. Its damage may involve all cardiac structures (pericardium, myocardium, valves, coronary arteries) and peripheral vessels, with variable onset modalities, in relation with dose, irradiation modality, contemporary administration of chemotherapic agents (in particular doxorubicin) and baseline patient's clinical condition [24]. Cardiovascular complications of radiation therapy may be acute and chronic. Some of these are true medical and/or surgery emergencies (cardiac tamponade, acute myocardial infarction, cardiac arrest). Others, mainly due to the progression of coronary atherosclerosis, remain silent for several years and produce during time severe coronary heart disease, even in absence of concomitant cardiovascular risk factors [25,26]. These vascular lesions correspond to intima hyperplasia and lumen wall collagen deposition, develop throughout a period of about 82 months and involve also carotid arteries, inducing stenosis particularly frequent at the level of bifurcation [27]. Of interest, heart valves are affected by collagen deposition due to radiation therapy and valvular stenosis or regurgitation may be severe, in particular at the level of mitral and aortic valves [25,27]. Pericardial involvement is, however, the most frequent anatomic consequence of radiation therapy. The right ventricle is more often and more extensively involved. The interval occurring between the radiation therapy and symptom onset is variable, ranging from 2 to 145 months and constrictive pericarditis, silent for several years, produces hemodynamic alterations which become clinically overt even long time after the cessation of x-ray exposition [28,29]. Importantly, the prevalence of all the abnormalities induced by the radiation therapy increased dramatically over time, making a strong argument for screening because they remained clinically unrecognized. In the past 30 years, radiation therapy has been deeply modified to reduce the total radiation dose administered and to better shield the cardiac structures. The prevalence of these cardiac abnormalities will, thus, realistically decrease in the patients treated more recently.

**Figure 1:**
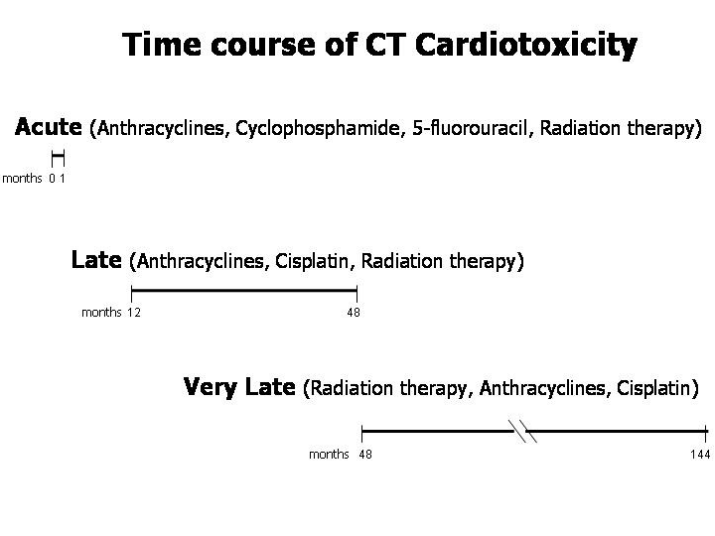
Schematic view of the time course of CT-related cardiotoxicity. CT = cancer therapy.

Table 2 shows schematically the different cardiovascular cellular and extracellular targets of CT [30-33]. It is worthy of note that some of these detrimental effects are actually used even beneficially in medicalized intracoronary stents to prevent restenosis after percutaneous coronary angioplasty. The cardiotoxicity of CT depends, however, on multiple factors which include the drug itself or radiation therapy but also the patient's individual characteristics. Dose of the administered drug during each session, cumulative dose, schedule of delivery, route of administration, combination with other drugs and with irradiation, sequence of administration of multiple medications should be taken carefully into account. Patient-related factors include age, pre-existing cardiovascular risk factors and previous cardiac disease, metabolic abnormalities and hypersensitivity to the given drug. In any case, CT-related cardiotoxicity may be acute (in particular immediately after a single dose of a course of anthracycline therapy), late, i.e. developing at long-term over time after completion of therapy, and very late, that is even 144 months (= 12 years) after the completion of radiation therapy or of multiple chemotherapies (Figure 1). This aspect is crucial to understand that the duration of cardiologic monitoring of patients undergoing CT needs to be very long and accurate.

**Table 2:** The main cardiovascular cellular and extracellular targets of CT

**Target Damage**	**Kind of CT**
Endothelial cells	Anthracyclines, Cyclophosphamide, 5-fluorouracil (TNF-α induced apoptosis), Monoclonal antibodies, Radiation therapy
Cardiomyocytes	Anthracyclines (atrophy and apoptosis), Cyclophosphamide, Cisplatin, 5-fluorouracil, Monoclonal antibodies (mitochondrial apoptosis), Radiation therapy
Smooth muscle cells	Anthracyclines Cytarabine, Cisplatin, 5-fluorouracil, Cytokines, Arsenic trioxide, Radiation therapy
Extracellular matrix	Anthracyclines, Cisplatin, 5-fluorouracil, Radiation therapy

## EKG and cardiotoxicity of CT

EKG represents the traditional support and completion to the clinical examination also for patients undergoing CT but EKG abnormalities are often non specific in this clinical setting. This is particularly true for anthracycline-induced acute toxicity, where non-specific ST-segment and T-wave changes, decreased QRS voltage and QT-interval prolongation have been described. Chronic anthracycline cardiotoxicity (even many years after the completion of therapy) manifests often as life-threatening arrhythmias [34]. 5-Fluorouracil has been demonstrated to be associated with EKG signs of myocardial ischemia while less frequent alterations include arrhythmias [35]. High dose of cyclophosphamide may cause malignant arrhythmias inducing sometimes fatal outcomes [36]. The most severe EKG alterations of interferon-α and interleukin-2 correspond to supra-ventricular and ventricular arrhythmias [37]. Effort EKG test may be useful to unmask coronary artery spasm induced by capecitabine, showing ST-segment elevation accompanied by angina [38]. Twenty-four-hour Holter monitoring and analysis of QT-interval dispersion may be important tools in patients undergoing CT, to detect number and severity of arrhythmias [39]. Increased QT-interval dispersion has been recently found to be a predictor of acute heart failure after therapy by cyclophosphamide [40] and to persist even in late survivors of childhood anthracycline treatment [41]. In relation with symptoms and signs of congestive heart failure, often developing during CT, it is, however, clear how EKG represents a limited tool for serial cardiologic assessment and early diagnosis of cardiac adverse effects and complications due to CT.

## Doppler-echo and cardiovascular toxicity of CT

Today, the widely used non-invasive method of monitoring cardiotoxicity of CT is certainly represented by Doppler echocardiography which allows to identify the main forms of cardiac involvement in cancer patient: LV (systolic and diastolic) dysfunction, valve heart disease, pericarditis and pericardial effusion, and carotid artery lesions.

**Figure 2:**
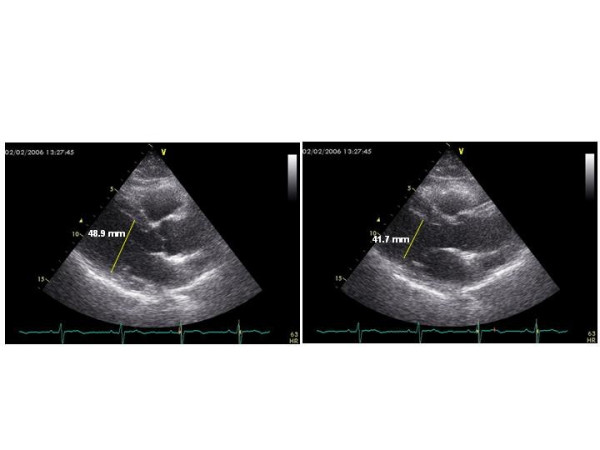
Acute LV systolic dysfunction in a 50-year old female patient affected by granulocytic sarcoma during CT by daunorubicine (50 mg/m^2^), cytarabine (30 mg/m^2^), etoposide 50 mg/m^2^. LV end-diastolic diameter (left panel) is 48.9 mm and LV end-systolic diameter (right panel) is 41.7 mm. Thus, endocardial fractional shortening is 14.7 % and EF = 37.9 %. EF = Ejection fraction, LV = Left ventricular.

**Figure 3:**
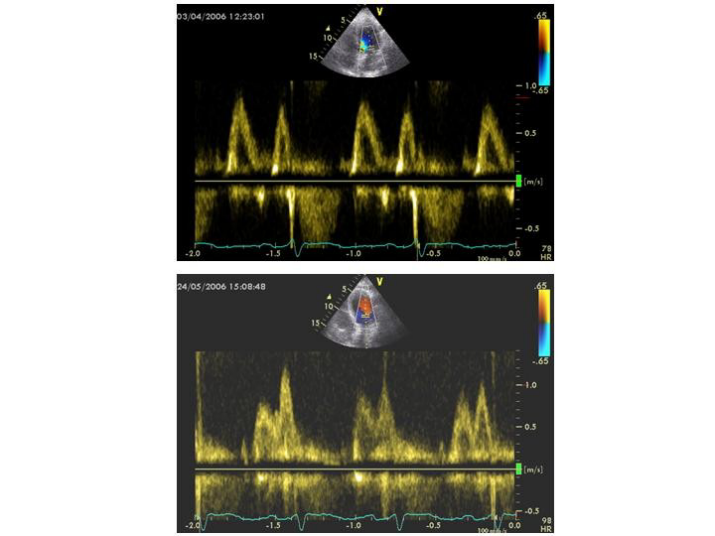
Doppler transmitral inflow pattern in a 58-year old female patient with acute myeloid leukemia before treatment (upper panel) and after DNR (= daunorubicine) 50 mg/m^2^, VP16 (= etoposide) 50 mg/m^2^, ARA-C (= cytarabine) 30 mg/m^2 ^(lower panel). Transmitral pattern, normal before treatment (transmitral E/A ratio = 1.12, deceleration time = 172 ms), shifts to pattern of LV abnormal relaxation, corresponding to grade I of LV diastolic dysfunction (E/A ratio = 0.70, deceleration time = 230 ms) after CT.

The form of CT-related cardiac involvement of the greatest interest and concern among oncologists and cardiologists is **LV dysfunction **which may progress until overt congestive heart failure [42]. LV endocardial fractional shortening and ejection fraction (EF) are the most common echocardiographic indexes of LV systolic function, used to quantify the degree of LV systolic dysfunction also in oncologic patients. Figure 2 shows the acute reduction of EF in a patient treated by an association of chemotherapic agents because of a granulocytic sarcoma. EF is, however, not very sensitive for the early diagnosis of preclinical heart disease. It is now recognized that the impairment of Doppler-derived diastolic indexes represents an early sign of LV dysfunction also in patients undergoing CT [43]. The diagnostic role of Doppler echocardiography for diagnosing LV dysfunction has been analyzed during and after anthracycline therapy. By using these drugs rare cases of acute and subacute cardiotoxicity resulting in acute heart failure were reported. A study performed to investigate possible acute effects in 88 patients with Hodgkin's lymphoma (average anthracycline dose = 174 mg/m^2 ^+ average mediastinum irradiation dose = 21 Gy = 21 Sv = 210.000 chest x-rays) did not show significant changes of M-mode derived LV end-diastolic diameter, end-systolic diameter and EF but identified regional wall motion abnormalities (hypokinesis) by 2D assessment [44]. The chronic cardiotoxicity often induces cumulative dose-related LV dysfunction and failure, which may be early detected by monitoring again Doppler diastolic indexes more than EF [45]. Early peak flow velocity to atrial peak flow velocity (E/A) ratio, deceleration time and isovolumic relaxation time were all impaired (grade I of diastolic dysfunction) in more than 50% of patients treated by anthracyclines, when EF was still normal [46]. Figure 3 depicts the shift of a baseline normal standard Doppler pattern of LV diastolic function to a pattern of LV abnormal relaxation (grade I of diastolic dysfunction) after DNR protocol (= daunorubicine + etoposide + cytarabine) in a woman affected by acute myeloid leukemia. The alteration of LV diastolic function occurs particularly in childhood, much more sensitive to the late cardiotoxicity of these chemotherapic agents [47,48]. In this context, myocardial performance index (= isovolumic contraction time + isovolumic relaxation time/ejection time), accurate marker of global LV function, may be particularly useful since it appeared significantly altered in 35 doxorubicin-treated children compared with that in 32 age-matched control subjects (0.42 ± 0.07 vs. 0.34 ± 0.06, p < 0.001) [49]. Serial echocardiograms performed in 101 patients with acute lymphoblastic leukemia and 83 survivors of Wilms tumor after a mean interval > 6 years since last anthracycline dose demonstrated that the most important predictor of worsening cardiac performance in childhood is the cumulative dose (> 240 mg/m^2^) [50]. The echocardiographic evaluation of cardiotoxicity due to drugs other than anthracyclines is less described. Echo monitoring appeared very useful to detect significant increase of both LV end-diastolic and end-systolic diameters/volumes, without decrease of fractional shortening, and reduction of transmitral E/A ratio in breast cancer patients undergoing high-dose (7 g/m^2^) cyclophoshamide [51]. Advanced breast cancer patients receiving epirubicin 90 mg/m^2 ^+ paclitaxel 200 mg/m^2 ^showed reduction of two-dimensional EF from baseline median value of 60% to median value of 50% after 8 courses [52]. Hypokinesis and decreased EF were observed after high-dose bolus of interleukin-2 and interferon-α in 16 patients treated for metastatic melanoma [53]. Of interest, while the imatinib mesylate administration may increase cardiac morbidity in advanced chronic myelogenous leukaemia treated by successive allogenic stem cell transplantation with busulphan/cyclophoshamide conditioning [54], the same drug appears able to induce a rapid, echocardiographically detected, reversion of Loeffler's endocarditis in early stage of clonal hypereosinophilic syndrome [55]. Echocardiography allowed also to unmask LV dysfunction in 39% of 41 patients, 5 years or more after receiving cardiac radiation for Hodgkin's disease or seminoma in remission [56]. The effect of radiation therapy (mediastinal irradiation for treatment of Hodgkin disease) on LV diastolic function was recently described by Doppler assessment: among the recruited 294 patients receiving at least 35 Gy (= 350.000 chest x-rays) to the mediastinum [57]. Worthy of note, patients with LV diastolic dysfunction had worse event-free survival than patients with normal function (hazard ratio 1.66, 95% CI 1.06–2.4) in a 3.2 year follow-up [57]. Data collected on the same population show that, compared with the Framingham Heart Study population, mildly reduced endocardial fractional shortening (<30%) was more common (36% vs. 3%), and age- and gender-adjusted LV mass was lower (90 ± 27 g/m vs. 117 g/m) in irradiated patients [58].

Also the documentation of **valvular heart disease, **developing as chronic CT of chemotherapic agents, concerns mainly anthracycline treatment. In a population of 305 patients (median age = 14 years), treated with a cumulative dose ranging 140–450 mg/m^2 ^for childhood malignancy, color flow Doppler detection of mitral regurgitation was evident in 34 patients (11.6%) compared with only 1.8% of a normal population of similar age (p < 0.0001) [59]. Since increased risk of left sided valvular regurgitation, in particular aortic regurgitation, was related to high-dose mediastinal radiation in 129 patients with Hodgkin's disease [60], echocardiographic screening is highly recommended in this clinical setting. This was confirmed in a retrospective study where, among patients treated with radiation therapy for Hodgkin lymphoma, there was statistically higher than expected rate of valve surgery and coronary revascularization procedures over the next 10 to 20 years [61] After a mean follow-up of 9.5 years the morbidity of valvular heart disease was about 2.8–2.9% in women who had undergone adjuvant radiotherapy for breast cancer [62]. The echocardiographic analysis of Heidenriech et al [58] observed that 60% of patients who had experienced irradiation (for Hodgkin's disease) more than 20 years earlier presented mild aortic regurgitation while 15% had moderate to severe aortic regurgitation. Despite the high prevalence of aortic valve disease in these patients, aortic regurgitation was rarely identified by physical examination. The echocardiographic features of radiation-associated valvular disease were described by Hering et al, who reported evidence of combined calcific transformation of the mitral valve, aortic valve and the aortic-mitral aponeurosis, i.e., the junction between the base of the anterior mitral leaflet and the aortic root [63].

**Figure 4:**
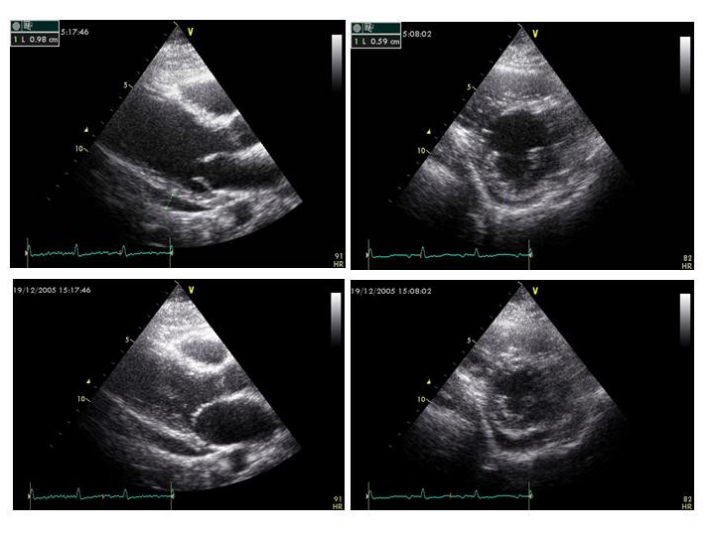
Detection of posterior pericardial effusion in a 28-year old female patient affected by non Hodgkin's lymphoma and treated by CHOP (Cyclophosphamide 750 mg/m 2 + Adriamycin 50 mg/m 2 + Vincristine 2 mg + Prednisone 100 mg) and previous mediastinal radiation therapy. The usual grading of pericardial effusion takes into account the diastolic separation between epicardium and pericardium: 1. small = < 10 mm corresponding to 300 ml, 2. moderate = 10–20 mm corresponding to 500 ml, 3. severe = > 20 mm corresponding to > 700 ml). In this patient the diastolic separation is < 10 mm (both in long and in short-axis vies), indicating mild pericardial effusion. Upper panel; visualization of diastolic pericardial effusion in parasternal long-axis (left panel) and short-axis view (right panel). Lower panel: visualization of systolic pericardial effusion in parasternal long-axis (left panel) and short-axis view (right panel).

Malignant pericardial disease is a serious but not uncommon problem seen in patients with cancer, usually due to metastatic spread of the underlying malignancy. **Pericarditis and pericardial effusion **can be also due to chemotherapy and, above all, to radiation therapy [64]. The patient may have a mild, subtle presentation, as often seen in the early stage of pericardial effusion, or may experience dramatic hemodynamic compromise, as occurring with cardiac tamponade and constrictive pericarditis. In all the cases, early diagnosis by echocardiography is fundamental. Observations of pericarditis and/or pericardial effusion induced by both acute and chronic anthracycline therapy were reported [44,65]. Radiation therapy is, however, the main cause of pericarditis and pericardial effusion, in an overall 30% incidence of clinically detectable heart injury after thoracic irradiation [66,67]. Acute pericarditis may occur early in the course of treatment, but constrictive or effusive pericarditis develop even months to years after therapy completion [67]. Acute and chronic pericarditis [68] and late appearance of chronic pericardial disease [69,70] after mediastinal radiation for Hodgkin lymphoma were reported in echocardiographic studies. In view of these findings, the size of the effusion has to be graded for appropriate management and comparison of repeated, serial examinations. The usual echocardiographic grading measures the echo free space in diastole [71] while the classification of Horowitz et al [72] takes into account the epicardium-pericardium separation in the different phases of the cardiac cycle (type A = no effusion, type B = systolic epicardium-pericardium separation corresponding to 3–16 ml, type C1 = systolic and diastolic separation corresponding to >16 ml, type C2 = systolic and diastolic separation with attenuated pericardial motion, type D = pronounced separation, with large echo-free space, type E = pericardial thickening [> 4 mm]). In addition, Doppler-echo signs of cardiac tamponade (exaggerated inspiratory variation of the two ventricles, right atrial and/or right ventricular collapse, left atrial and/or left ventricular collapse, inferior cava plethora, abnormal increased respiratory variation in transvalvular blood flow velocities) should be promptly diagnosed [71]. The search of pericardial effusion and thickening should be performed by multiple views (including sub-costal view), in order to explore the entire pericardial status. Figure 4 shows a mild pericardial effusion in a woman affected by non Hodgkin's lymphoma and treated by a CHOP association and previous mediastinal radiation therapy.

Asymptomatic **carotid arterial disease **occurs frequently in young patients following head and neck radiation therapy. Doppler-echo scan of carotid arteries allowed to detect increased intima-media thickness in 24% of 42 survivors of Hodgkin lymphoma who had undergone radiation therapy more than 5 years earlier and one patient had greater than 70% stenosis of both common carotid arteries [73]. This findings were recently confirmed in 12/21 patients, irradiated for Hodgkin disease, non Hodgkin lymphoma and seminoma, who developed atherosclerotic carotid vascular disease [74]. In this view, the observation that survivors of childhood Hodgkin disease undergoing mantle irradiation are at increased risk of stroke (RR = 5.62, 95% CI = 2.59 to 12.25, p < 0.0001) is not unexpected [75].

All together, these findings highlight the ultrasound ability to unmask cardiovascular abnormalities in symptomatic as well as in asymptomatic patients undergoing CT. It emerges also by our retrospective experience including 148 echocardiographic observations from patients referring to our echo-lab for hematologic malignancies and showing CT-related abnormalities of LV function, pericardium and heart valves [76].

## Stress echocardiography and detection of cardiotoxicity

Dobutamine stress echocardiography was tested in several studies to detect subclinical abnormalities of LV function induced by anthracycline cardiotoxicity [77-81] but the findings of these studies appear insufficient or controversial. In two reports [77,78] low-dose dobutamine, performed to oncologic patients before, during and after 6 months following chemotherapy, was able to unmask a reduced contractile reserve only in patients who had already depressed LV systolic function and standard Doppler pattern of LV abnormal relaxation at rest. In the experience of Lanzarini et al [80], young oncologic patients, who had undergone high-dose anthracycline therapy (>400 mg/m^2^) and had normal LV structure and function at rest, did not show significant modifications during a modified/accelerated dobutamine protocol (achievement of the maximal dose of 15 mg/Kg/min in a short time). On the other hand, Hamada et al, by using high-dose dobutamine (30 mg/Kg/min), evidenced alteration of posterior wall thickness, fractional shortening and transmitral E/A ratio in patients treated by high-dose anthracycline and free of cardiologic symptoms (81).

Stress echocardiography, an optimal tool to unmask coronary artery disease in the general population, remains important also in patients treated by some chemotherapic agents (e.g., capecitabine) and in particular by radiation therapy, which is able to provoke accelerated coronary atherosclerosis. Experiences dealing with the use of stress echocardiography in this clinical setting are, however, lacking until now.

## Advanced ultrasound tools for early detection of cardiotoxicity

Some high-tech ultrasound tools as Integrated Backscatter (IBS) and Tissue Doppler have been used with the aim to identify subclinical alterations of both LV structure and function in patients treated by CT.

Analysis of acoustic intensity of the backscattered signal consists of the omni-directional scattering energy redirected back to the transducer, with different intensity according to the density of the reflecting tissue [82]. IBS, which reliably identifies in vivo variations in the regional extent of myocardial fibrosis [83,84], was successfully used in 28 oncologic patients under HDLV5FU chemotherapy. In these patients the magnitude of both anterior and posterior cardiac IBS values significantly decreased at 48^th ^hour of treatment compared with 0^th ^hour but returned near the baseline values at day 15 (p = 0.003), suggesting a reversible, acute 5-fluorouracile toxicity [85]. In addition, in relation with the recognized diastolic-to-systolic variation of IBS signal [86], cyclic-variation-IBS of posterior wall appeared decreased in 32 patients with non Hodgkin's lymphoma after 300 mg/m^2 ^of doxorubicin, this decrease being higher in patients treated by moderate and high-dose than in those undergoing low dose [87].

**Figure 5:**
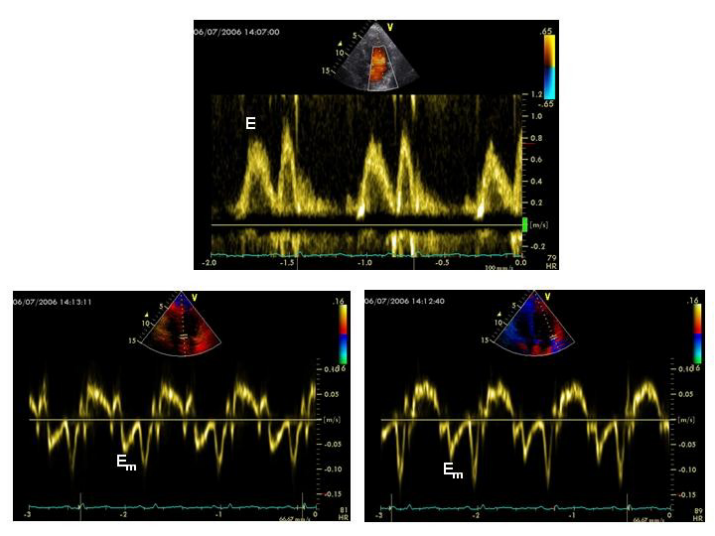
Sample of the combination of standard Doppler mitral inflow and Tissue Doppler of septal and lateral mitral annulus in a 38-year male patient affected by acute promyelocytic leukemia undergoing CT by idarubicine (12 mg/m^2^) + trans-retinoic acid (45 mg/m^2^). In the upper panel transmitral E/A ratio is > 1 (E peak velocity = 0.78 m/s); In the lower panels Tissue Doppler derived Em peak velocity is 0.07 m/s (left panel) and 0.08 m/s (right panel) respectively, determining an average Em = 0.075 m/s. Thus, E/Em ratio is = 10.4, indicating an initial increase of LV filling pressure (normal values of E/Em ratio < 8, see Ref # 90). E = transmitral early diastolic peak velocity, Em = Tissue Doppler derived myocardial early diastolic peak velocity.

IBS requires time-expensive, off-line analysis and normalization for the pericardial interface, inducing possible artifacts. In contrast, pulsed Tissue Doppler may be easily performed during a standard Doppler echocardiographic examination by using a high-pass filter and placing a sample volume on a given region of interest [88]. It has been successfully applied in several clinical setting and appears reliable to provide quantitative information on myocardial diastolic relaxation and systolic performance [88]. Tissue Doppler of LV lateral mitral annulus has a recognized prognostic role [89] and, in combination with standard Doppler mitral inflow, provides accurate information about the degree of LV filling pressure [90]. Early changes in LV myocardial function of oncologic patients were found by pulsed Tissue Doppler of multiples LV sites (mitral annulus, basal LV lateral and basal LV posterior wall) early after (1–3 months) and, more accentuated, at the late control (3.5 ± 0.6 years) after the completion of doxorubicin therapy (cumulative dose = 211 ± 82 g/m^2^). In these patients also myocardial systolic dysfunction was evident, in presence of normal EF [91,92]. Similar findings were observed also in survivors of childhood cancer treated with anthracyclines [93], while no experience is available about the use of Tissue Doppler in patients treated by other chemotherapic agents or radiation therapy. Figure 5 depicts standard Doppler mitral inflow pattern and Tissue Doppler of septal and lateral mitral annulus in a in a 38-year male patient affected by acute pro-myelocitic leukemia undergoing idarubicine and trans-retinoic acid: transmitral E/A ratio is normal (>1) but the ratio between transmitral E and the average value of Tissue Doppler derived Em (Septal Em + Lateral Em/2) is = 10.4 (normal values < 8), indicating an initial increase of LV filling pressure.

**Figure 6:**
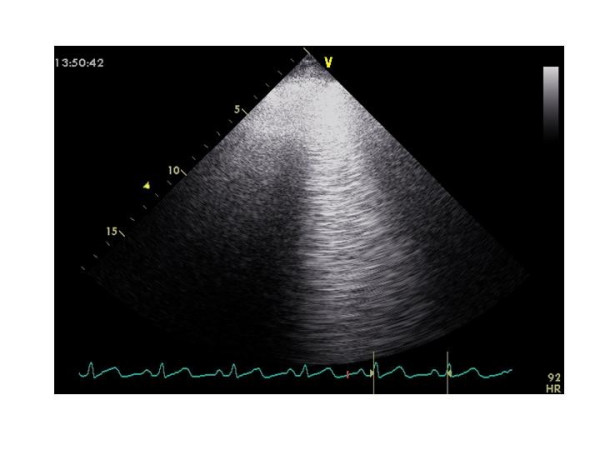
Ultrasound evidence of lung water ("comet tail") of V inter-costal space in a 58-year old male patient with pulmonary microcitoma after CT including carboplatinum, etoposide and radiation therapy.

An useful tool to identify early markers of pulmonary congestion following to heart failure could be represented by the ultrasound "lung comet", an echocardiographic sign detectable with the ultrasound scan of anterior and lateral chest (from the second to the fifth intercostal space). It is visualized as multiple comet-tails fanning out from the lung surface [94]. It originates from water-thickened or fibrotic interlobular septa and allows a bedside distinction between pulmonary edema and obstructive lung disease [95,96]. "Lung comet" can be particularly appealing for identifying and quantifying extravascular lung water, a hinge parameter in the serial evaluation of the cardiologic patient with heart failure [97]. Of interest, this sign is detectable before the onset of pulmonary rales by lung auscultation, does not need sophisticated technology and may be performed at bedside, even with hand-held echocardiographic machines of low cost and limited tools. Specific applications of ultrasound "lung comet" for CT monitoring has not yet reported but in our clinical practice it can be visualized particularly in oncologic patients who experienced radiation therapy (Figure 6). In these cases it might be considered a sign of alveolar-capillary membrane distress (ARDS-like syndrome), already demonstrated by other imaging techniques and due to acute, deterministic, combined radiation and chemo-therapy [98,99].

## Conclusion

A large amount of data demonstrate how Doppler echocardiography represents the election tool not only for baseline cardiologic screening but also for follow-up of the oncologic patients during and after the completion of CT. In relation with its low cost, feasibility and reliability to diagnose LV systolic and diastolic dysfunction, regional wall motion abnormalities, valve disease, pericardial abnormalities and carotid lesions, the ultrasound examination appears very useful to identify and monitor CT-related cardiac conditions over time. This is really crucial when using some chemotherapic agents as anthracyclines, which have early but, most importantly, late and very late, cumulative cardiac toxicity. The late cardiac toxicity may be stopped or at least blunted by a prompt pharmacological intervention. The echocardiographic monitoring appears even indispensable after radiation therapy, whose detrimental effects may appear several years after the end of irradiation. Under these circumstances and given the high likelihood of diagnosing significant cardiovascular pathologies, an ultrasound screening 10 years after irradiation is recommendable also in patients who remain asymptomatic and should be extended to the exploration of carotid arteries in patients undergoing mantle irradiation. In all the cases the performance of Doppler-echo examination should be very flexible, prone to the assessment of multiple parameters of LV systolic and diastolic function (including Tissue Doppler of LV mitral annulus), to the detection and grading of valvular heart disease, to the visualization of overall pericardium by multiple views and even to the measure of carotid intima-media thickness (Table 3). Bio-humoral markers of cardiac function (BNP and NT-proBNP) could provide further support during the follow-up and indicate the right timing for repetition of Doppler echocardiographic examination in subgroups at risk for heart failure (91). This will be useful to anticipate treatment and decide the most appropriate pharmacological therapy for congestive heart failure at its early onset.

**Table 3:** The 10 commandments for optimal Doppler-echo scan of oncologic patients

**1**	Quantify LV geometry (wall thickness, cavity diameters, relative wall thickness, LV mass).
**2**	Search regional wall motion abnormalities.
**3**	Estimate ejection fraction by 2D apical views if wall motion abnormalities are evident.
**4**	Analyze standard Doppler indexes of LV diastolic function.
**5**	Record pulsed Tissue Doppler of mitral annulus for detection of increasing LV filling pressure.
**6**	Explore structural and functional valve features, in particular mitral and aortic valves.
**7**	Visualize pericardium in all ultrasound views (including sub-costal), particularly in patients at high risk (anthracycline, irradiation therapy).
**8**	Search ultrasound "comet tail" in patients at risk (anthracycline, irradiation therapy).
**9**	Scan carotids in patients treated by head and neck irradiation.
**10**	Perform stress echocardiography if coronary artery disease is suspected.

## Competing interests

The author(s) declare that they have no competing interests.
